# Association between introduction of 13-valent pneumococcal conjugate vaccine and burden of hospitalized adult pneumococcal disease in Taiwan – a retrospective database study

**DOI:** 10.1017/S0950268826101411

**Published:** 2026-06-26

**Authors:** Chi-Chuan Wang, Ting-An Yen, Jhong-Lin Wu, Ling-Ya Huang, See-Hwee Yeo, Dony Patel, Cindy Lim, Hung-Wei Lin, Eriko Yamada, Isaya Sukarom

**Affiliations:** 1 School of Pharmacy, College of Medicine, National Taiwan University, Taipei, Taiwan; 2 Graduate Institute of Clinical Pharmacy, College of Medicine, National Taiwan University Hospital, Taipei, Taiwan; 3 Department of Pharmacy, National Taiwan University Hospital, Taipei, Taiwan; 4 Department of Pediatric, National Taiwan University Hospital, Taipei, Taiwan; 5 Department of Emergency Medicine, National Taiwan University Hospital, Taipei, Taiwan; 6 Real World Solutions, IQVIA Solutions Asia, Singapore, Singapore,; 7 Global Database Studies, Real World Solutions, IQVIA, London, UK; 8 Real World Solutions, IQVIA Solutions Taiwan, Taipei, Taiwan; 9 Global Medical and Scientific Affairs, MSD Singapore, Singapore, Singapore; 10 Regional Outcomes Research, MSD Thailand, Bangkok, Thailand

**Keywords:** invasive pneumococcal disease, pneumococcal conjugate vaccine, pneumonia, Taiwan, incidence rate, healthcare resource utilization, case fatality rate, costs

## Abstract

This study estimated the incidence rate (IR), case fatality rate (CFR), healthcare resource utilization, and costs of pneumococcal disease before and after the introduction of the 13-valent pneumococcal conjugate vaccine (PCV13) into Taiwan’s childhood immunization programme (CIP) in 2015. Adults aged ≥18 years hospitalized with invasive pneumococcal disease (IPD) and pneumonia from 2011 to 2019 were identified from the Taiwan National Health Insurance Research Database. Baseline and trend of IRs were compared between pre-introduction (2011–2014) and post-introduction (2016–2019) of PCV13 using interrupted time series analyses and incidence rate ratio (IRR). Annual IRs for IPD and pneumonia ranged from 1.59 to 1.82 and 3.91 to 4.47 episodes per 100,000 person-years in the pre-PCV13 period, respectively. In the post-PCV13 period, they ranged from 1.23 to 1.37 and 3.20 to 3.95 episodes per 100,000 person-years, respectively. There was a significantly lower baseline IR in the post-PCV13 period for both IPD (IRR = 0.76; 95% confidence interval [CI]: [0.63, 0.93]) and pneumonia (IRR = 0.87; 95% CI: [0.77, 0.98]). However, a marginal increase in the trend of IR was observed during the post-PCV13 period for both IPD (IRR = 1.02; 95% CI: [1.00, 1.03]) and pneumonia (IRR = 1.02; 95% CI: [1.01, 1.03]). Estimated CFRs were lower in the post-PCV13 period for both diseases. The clinical burden remains substantial after the introduction of PCV13 into Taiwan’s CIP.

## Background

*Streptococcus pneumoniae* is the causative agent of invasive pneumococcal disease (IPD), including bacteraemia, meningitis, and pneumonia [1]. According to statistics from the Taiwan National Infectious Disease Statistics System, the annual incidences of IPD among adults aged 18–49 years and 50–64 years were 1.1 and 3.2 cases per 100,000 population from 2008 to 2012, respectively [2]. The incidences of IPD among adults aged 65–74 years and ≥ 75 years were 7.3 and 14.0 cases per 100,000 population during the same period, indicating the higher risk of IPD in the elderly [2]. The burden of pneumonia is similarly high in Taiwan [3]. It was the third leading cause of death in 2018 [4]. Annual pneumonia hospitalizations ranged between 346,144 and 407,016 cases from 2011 to 2016, with the rate of hospital admissions being higher in the elderly aged ≥65 years [5, 6].

There are currently two kinds of pneumococcal vaccines widely used across countries, namely the pneumococcal polysaccharide vaccine and the pneumococcal conjugate vaccine (PCV). These vaccines contain polysaccharides from different serotypes of *S. pneumoniae* and promote the development of anti-capsular antibodies to the type-specific capsular polysaccharide antigens, thereby conferring immunity against infection by *S. pneumoniae* [7]. In Taiwan, the 23-valent pneumococcal polysaccharide vaccine (PPSV23) has been recommended for the elderly since 2008, while the 13-valent pneumococcal conjugate vaccine (PCV13) was introduced into the childhood immunization programme (CIP) in 2015 [8]. The age recommendation for PPSV23 administration in the elderly was also lowered from 75 to 71 years in 2022 [9].

Based on a previous study conducted in the United States, the introduction of PCV13 in children could have an indirect protective effect among adults, a phenomenon known as the herd effect [10]. However, there are also some European studies which showed that the herd effect is negligible [11, 12]. These observations may be explained by geographical differences in the prevalence of circulating pneumococcal serotypes and vaccine coverage rates [13]. PCV13 coverage in Taiwanese children rose quickly from 31.9% in 2013 to 95.8% in 2017, but little is known about the association between PCV13 inclusion into the CIP and the burden of pneumococcal disease in adults [14, 15]. Therefore, this study aimed to quantify the clinical and economic burden associated with PCV13 by examining the hospitalized incidence rate (IR), case fatality rate (CFR), healthcare resource utilization (HCRU), and costs associated with IPD and pneumonia in adults, before and after the introduction of PCV13 into Taiwan’s CIP.

## Methods

### Study design and population

A nationwide, retrospective population-based study was conducted using the National Health Insurance Research Database (NHIRD), a national claims database covering 99% of the Taiwanese population [16]. Data were extracted from adult patients (≥18 years) hospitalized with IPD or pneumonia between 1 January 2011 and 31 December 2019. IPD and pneumonia episodes were identified based on pneumococcal-specific International Classification of Disease (ICD) codes (the ICD codes are provided in Supplementary Table 1).

The date of the first hospitalization indicated the start of a disease episode. The time period or gap between the end of one hospitalization (discharge) and the start of the next hospitalization was used to define episodes. A disease episode was considered new if a subsequent hospitalization (with the same diagnosis codes) occurred after a time gap of at least 90 days, while those occurring within 90 days were assumed to be related to the earlier episode and were collapsed into a single episode. This approach is consistent with a previously published study, and the time gap has been validated by clinical experts [17].

### Outcome measurements

The primary outcomes were IR, CFR, HCRU, and costs of hospitalized IPD and pneumonia episodes. IR was calculated as the number of new disease episodes divided by total person-years at risk of pneumococcal disease. CFR was estimated as the proportion of in-hospital deaths among disease episodes. In-hospital cause-related mortality was identified from the National Death Registry and defined as any death occurring during hospitalization or within a 3-day window after discharge with ICD codes corresponding to sepsis, meningitis, pneumonia, acute respiratory failure, or pulmonary heart diseases [18]. These causes of death were considered to be related to IPD or pneumonia. HCRU included the total length of hospitalization, as well as the number of inpatient, outpatient, and emergency department (ED) visits. Costs included inpatient, outpatient, outpatient pharmacy, and ED costs, and were expressed in New Taiwan Dollars (NTD).

### Statistical analyses

Study findings were summarized with descriptive statistics. Continuous variables were reported using means, standard deviations, medians, and interquartile ranges, and compared using Student’s t-test or Wilcoxon rank-sum test. Quarterly time trends of IR were assessed with interrupted time series analyses (ITSAs) based on a Poisson or negative binomial regression model for each disease. Incidence rate ratios (IRRs) were computed by exponentiating the relevant beta coefficients to compare the baseline and trends of IRs between time periods. Baseline IR referred to the IR at the start of the pre- and post-PCV13 periods, while the IR trend referred to the gradient/slope of the IRs during the pre- and post-PCV13 periods.

Study outcomes were compared between pre-PCV13 (2011–2014) and post-PCV13 (2016–2019) periods. The year 2015 was considered the transition period and examined separately since it was the year of PCV13 introduction into Taiwan’s CIP. Analyses stratified by age groups (18 to <65, and ≥65 years) were also performed.

Sensitivity analyses were conducted with different definitions of hospitalized IPD and pneumonia. Disease episodes were identified with both pneumococcal-specific and non-specific ICD codes (the non-specific ICD codes are provided in Supplementary Table 1) to examine how the estimation of IRs and ITSA results might differ from those of the primary analyses. For instance, ICD codes for non-specific and all-cause pneumonia were also used in the sensitivity analyses.

All analyses were performed using SAS version 9.2 (SAS Institute Inc., North Carolina, United States of America), and a *P* value <0.05 was considered statistically significant.

## Results

A total of 10,575 hospital episodes were identified between 2011 and 2019, comprising 3,001 IPD and 7,574 pneumonia episodes.

### IPD incidence

**Figure 1.**
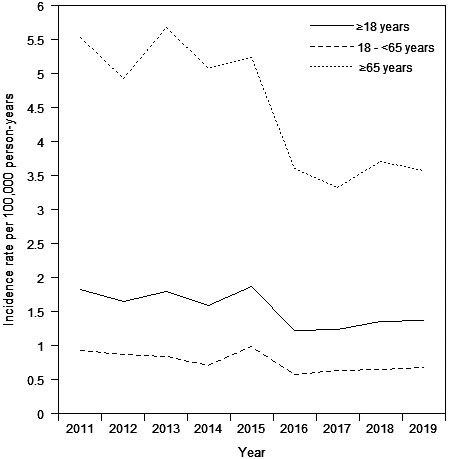
Trend in annual incidence of hospitalized invasive pneumococcal disease in adults from 2011 to 2019. Overall IR for hospitalized IPD was 1.54 episodes per 100,000 person-years, and it was approximately six times higher in patients ≥65 years than those 18 to <65 years. Annual IPD IRs were generally lower in the post-PCV13 period when compared with pre-PCV13 period. Figure 1. long description.

**Figure 2.**
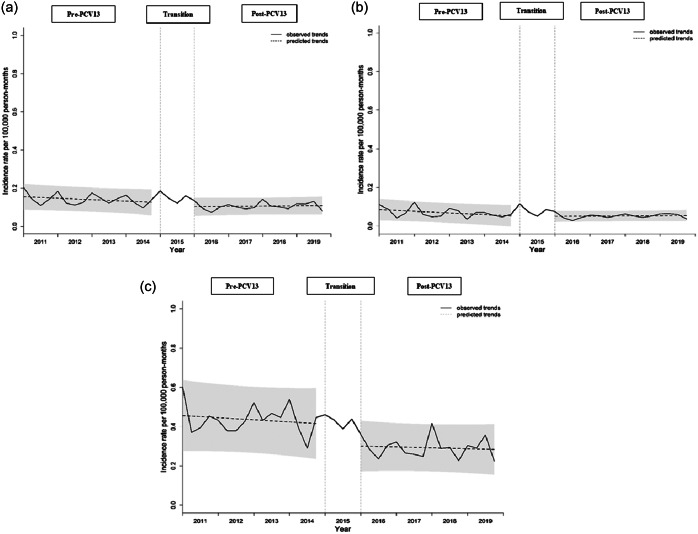
Trend in quarterly incidence of hospitalized invasive pneumococcal disease in adults aged (a) ≥18 years, (b) 18 to <65 years, and (c) ≥65 years from 2011 to 2019. (a) For adults aged ≥18 years, there was a significantly lower baseline level of IR in the post-PCV13 period (IRR = 0.76; 95% CI: [0.63, 0.93]), compared with the pre-PCV13 period, and a significant increase in the IR trend was observed in the post-PCV13 period (IRR = 1.02; 95% CI: [1.00, 1.03]). (b) For adults aged 18 to <65 years, baseline IRs were comparable between the pre- and post-PCV13 periods (IRR = 0.90; 95% CI: [0.66, 1.23]), and a significant increase in the IR trend was observed in the post-PCV13 period (IRR = 1.04; 95% CI: [1.01, 1.06]). (c) For adults aged ≥65 years, baseline IR was significantly lower in the post-PCV13 period (IRR = 0.68; 95% CI: [0.53, 0.87]), compared with the pre-PCV13 period, and there was no significant change in trend between the two periods (IRR = 1.00; 95% CI: [0.98, 1.02]). Figure 2. long description.

The overall IR for hospitalized IPD was 1.54 episodes per 100,000 person-years (Figure 1, numerical data further provided in Supplementary Table 2). The IR was approximately six times higher in patients ≥65 years than in those 18 to <65 years (4.45 vs. 0.76 episodes per 100,000 person-years, respectively). Annual IPD IRs were generally lower in the post-PCV13 period (ranging between 1.23 and 1.37 episodes per 100,000 person-years), when compared with the pre-PCV13 period (ranging between 1.59 and 1.82 episodes per 100,000 person-years). According to ITSA results for quarterly IPD IRs, there was a significantly lower baseline level of IR in the post-PCV13 period (IRR = 0.76; 95% confidence interval [CI]: [0.63, 0.93]), compared with the pre-PCV13 period (Figure 2a and Table 1). Conversely, a marginal increase in the IR trend was observed in the post-PCV13 period (IRR = 1.02; 95% CI: [1.00, 1.03]).

**Table 1.** IRRs of baseline IR and trend of IR in the post-PCV13 period compared to the pre-PCV13 period Table 1. long description.

Condition	Baseline IR			Trend of IR		
	IRR	95% CI	*p* value	IRR	95% CI	*p* value
**IPD**						
≥ 18 years	0.76	(0.63, 0.93)	0.007^a^	1.02	(1.00, 1.03)	0.045^a^
18 to <65 years	0.90	(0.66, 1.23)	0.507	1.04	(1.01, 1.06)	0.010^a^
≥ 65 years	0.68	(0.53, 0.87)	0.002^a^	1.00	(0.98, 1.02)	0.831
**Pneumonia**						
≥ 18 years	0.87	(0.77, 0.98)	0.019^a^	1.02	(1.01, 1.03)	<0.001^a^
18 to <65 years	0.90	(0.74, 1.09)	0.291	1.03	(1.01, 1.04)	0.002^a^
≥ 65 years	0.83	(0.71, 0.97)	0.016^a^	1.01	(1.00, 1.03)	0.045^a^

a *p* value <0.05.

Abbreviations: CI, confidence interval; IPD, invasive pneumococcal disease; IR, incidence rate; IRR, incidence rate ratio; PCV13, 13-valent pneumococcal conjugate vaccine.

Baseline IPD IRs were comparable between the pre- and post-PCV13 periods among patients aged 18 to <65 years (IRR = 0.90; 95% CI: [0.66, 1.23]), but a significant increase in IR trend was observed during the post-PCV13 period (IRR = 1.04; 95% CI: [1.01, 1.06]) (Figure 2b and Table 1). Although baseline IR was significantly lower for those ≥65 years in the post-PCV13 period than in the pre-PCV13 period (IRR = 0.68; 95% CI: [0.53, 0.87]), there was no statistically significant change in trend between the time periods (IRR = 1.00; 95% CI: [0.98, 1.02]) (Figure 2c and Table 1).

### Pneumonia incidence

**Figure 3.**
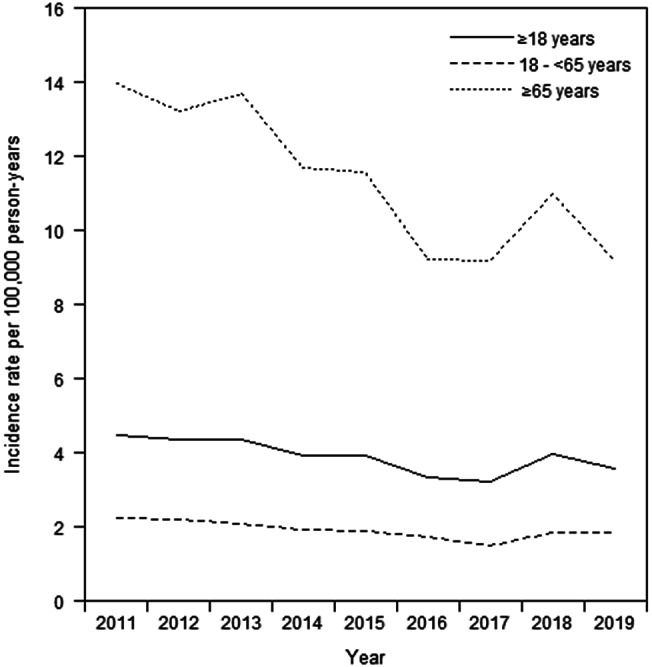
Trend in annual incidence of hospitalized pneumonia in adults by age groups from 2011 to 2019. Overall IR for hospitalized pneumonia was 3.89 per 100,000 person-years, and it was approximately six times higher in patients ≥65 years than those 18 to <65 years. Annual pneumonia IRs were generally lower in the post-PCV13 period when compared with pre-PCV13 period. Figure 3. long description.

**Figure 4.**
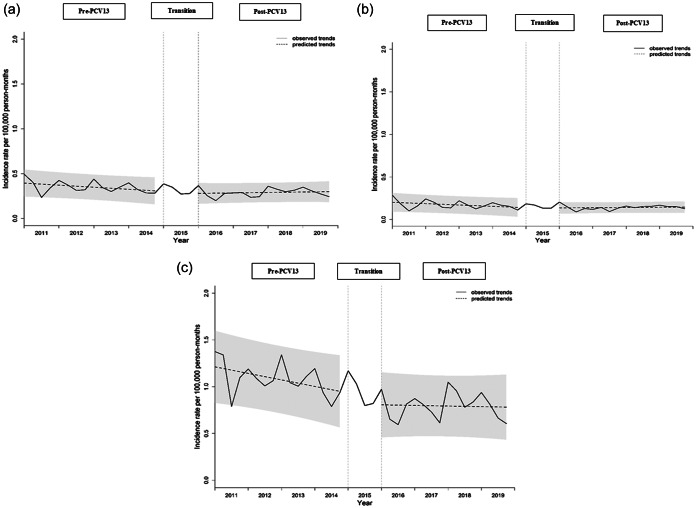
Trend in quarterly incidence of hospitalized pneumonia in adults aged (a) ≥18 years, (b) 18 to <65 years, and (c) ≥65 years from 2011 to 2019. (a) For adults aged ≥18 years, there was a significantly lower baseline level of IR in the post-PCV13 period (IRR = 0.87; 95% CI: [0.77, 0.98]), compared with the pre-PCV13 period, and a significant increase in the IR trend was observed in the post-PCV13 period (IRR = 1.02; 95% CI: [1.01, 1.03]). (b) For adults aged 18 to <65 years, baseline IRs were comparable between the pre- and post-PCV13 periods (IRR = 0.90; 95% CI: [0.74, 1.09]), and a significant increase in the IR trend was observed in the post-PCV13 period (IRR = 1.03; 95% CI: [1.01, 1.04]). (c) For adults aged ≥65 years, baseline IR was significantly lower in the post-PCV13 period (IRR = 0.83; 95% CI: [0.71, 0.97]), compared with the pre-PCV13 period, and a significant increase in the IR trend was observed in the post-PCV13 period (IRR = 1.01; 95% CI: [1.00, 1.03]). Figure 4. long description.

The overall IR for hospitalized pneumonia was 3.89 episodes per 100,000 person-years (Figure 3, numerical data further provided in Supplementary Table 2). The IR was estimated to be about six times higher in patients ≥65 years than in those 18 to <65 years (11.27 vs. 1.91 episodes per 100,000 person-years, respectively). The ranges of annual pneumonia IRs for patients aged ≥18 years, 18 to <65 years, and ≥ 65 years were generally lower in the post-PCV13 period (3.20–3.95, 1.49–1.85 and 9.16–10.99 episodes per 100,000 person-years, respectively), compared with pre-PCV13 period (3.91–4.47, 1.93–2.21, and 11.69–13.96 episodes per 100,000 person-years, respectively). Similar to IPD, although the baseline pneumonia IR for patients ≥18 years was significantly lower in the post-PCV13 period (IRR = 0.87; 95% CI: [0.77, 0.98]), there was a significant increase in the IR trend (IRR = 1.02; 95% CI: [1.01, 1.03]) (Figure 4a and Table 1).

When stratified by age groups, baseline pneumonia IRs were comparable between pre- and post-PCV13 periods for adults 18 to <65 years (IRR = 0.90; 95% CI: [0.74, 1.09]) (Figure 4b and Table 1), while there was a significantly lower baseline pneumonia IR among those ≥65 years in the post-PCV13 period (IRR = 0.83; 95% CI: [0.71, 0.97]) (Figure 4c and Table 1). Significant increases in IR trends were observed in both age groups 18 to <65 years and ≥ 65 years (IRR = 1.03; 95% CI: [1.01, 1.04] and IRR = 1.01; 95% CI: [1.00, 1.03], respectively) during the post-PCV13 period.

### Case fatality rate

The overall CFRs of hospitalized IPD and pneumonia were 17.23% and 7.00%, respectively (Table 2). In addition, CFR among patients aged ≥65 years was about 2.5–3 times higher than that among patients aged 18 to <65 years for both IPD (22.30% vs. 9.31%) and pneumonia (9.31% vs. 3.34%). CFRs of IPD for patients aged ≥65 years and 18 to <65 years were 21.22% and 7.89% in the post-PCV13 period, respectively. These were lower when compared with CFRs in the pre-PCV13 period for the respective age groups (23.45% and 9.81%). Similarly, CFRs of pneumonia were lower in the post-PCV13 period than in the pre-PCV13 period in patients aged ≥65 years (7.40% vs. 10.38%) and 18 to <65 years (2.55% vs. 4.04%).

**Table 2.** Case fatality rates by pneumococcal disease, age groups, and PCV13 periods Table 2. long description.

Condition	Overall			Pre-PCV13 period			Transition			Post-PCV13 period		
	Cases (N)	Deaths (*n*)	CFR (%)	Cases (N)	Deaths (*n*)	CFR (%)	Cases (N)	Deaths (*n*)	CFR (%)	Cases (N)	Deaths (N)	CFR (%)
**IPD**												
≥18 years	3,001	517	17.23	1,458	264	18.11	405	69	17.04	1,138	184	16.17
18 to <65 years	1,171	109	9.31	571	56	9.81	169	19	11.24	431	34	7.89
≥65 years	1,830	408	22.30	887	208	23.45	236	50	21.19	707	150	21.22
**Pneumonia**												
≥18 years	7,574	530	7.00	3,632	286	7.87	847	72	8.50	3,095	172	5.56
18 to <65 years	2,935	98	3.34	1,435	58	4.04	325	10	3.08	1,175	30	2.55
≥65 years	4,639	432	9.31	2,197	228	10.38	522	62	11.88	1,920	142	7.40

Abbreviations: CFR, case fatality rates; IPD, invasive pneumococcal disease; PCV13, 13-valent pneumococcal conjugate vaccine.

### Healthcare resource utilization

There were no significant differences in the median length of hospitalization per inpatient visit between pre-PCV13 and post-PCV13 periods for both IPD (12 vs. 11 days; *p* value = 0.117) (Table 3) and pneumonia (9 vs. 8 days; *p* value = 0.422) (Table 4). However, for the age group ≥65 years, it was significantly shorter in the post-PCV13 period for IPD than the pre-PCV13 period (11 vs. 13 days; *p* value = 0.016). The total costs (inpatient, ED, and outpatient) were lower for IPD (NTD 102,209,008 vs. 185,975,875) and pneumonia (NTD 192,199,394 vs. 312,572,871) in the post-PCV13 period, compared with the pre-PCV13 period. The average total costs per episode were also marginally lower in the post-PCV13 period than in the pre-PCV13 period for both IPD (NTD 122,406 vs. 127,555) and pneumonia (NTD 83,420 vs. 86,061). In addition, lower average total costs per episode were also observed in the post-PCV13 period among patients 18 to <65 years for pneumonia (NTD 60,673 vs. 64,184), as well as those ≥65 years for IPD (NTD 124,124 vs. 138,646) and pneumonia (NTD 97,119 vs. 100,350). Between the two age groups, patients aged ≥65 years generally had higher HCRU and higher costs per episode than those aged 18 to <65 years.

**Table 3.** IPD-associated HCRU and costs during pre-PCV13 and post-PCV13 periods, by age groups Table 3. long description.

	≥18 years			18 to <65 years			≥65 years		
	Pre-PCV13 (*n* = 1,458)	Post-PCV13 (*n* = 835)	*p* value	Pre-PCV13 (*n* = 571)	Post-PCV13 (*n* = 316)	*p* value	Pre-PCV13 (*n* = 887)	Post-PCV13 (*n* = 519)	*p* value
**Healthcare resource utilization**									
Inpatient									
Number of visits^b^, *n*	1,561	894	–^h^	597	336	–	964	558	–
Length of hospitalization per visit (days)									
Median (Q1, Q3)	12 (7, 20)	11 (7, 19)	0.117	10 (6, 17)	11 (7, 18)	0.117	13 (8, 22)	11 (7, 19)	0.016^a^
Total length of hospitalization^c^	25,678	13,642	–	8,338	4,955	–	17,340	8,687	–
Per episode									
Average number of visits	1.07	1.07	–	1.05	1.06	–	1.09	1.08	–
Average length of hospitalization (days)	17.61	16.34	–	14.60	15.68	–	19.55	16.74	–
Outpatient									
Number of visits^d^, *n*	281	236	–	133	127	–	148	109	–
Average number of visits per episode	0.19	0.28	–	0.23	0.40	–	0.17	0.21	–
ED									
Number of visits^e^, *n*	119	88	–	40	34	–	79	54	–
Average number of visits per episode	0.08	0.11	–	0.07	0.11	–	0.09	0.10	–
**Costs**									
Inpatient									
Per visit									
Median (Q1, Q3)	61,970.50 (33,762.00, 136,881.00)	66,991.00 (36,848.00, 132,740.00)	0.127	54,216.00 (29,868.00, 111,919.00)	65,364.00 (35,174.00, 132,229.00)	0.006^a^	69,261.00 (38,607.00, 158,059.00)	68,763.00 (37,746.00, 132,740.00)	0.268
Total inpatient costs	184,686,531	101,005,691	–	62,532,727	37,207,503	–	122,153,804	63,798,188	–
Average inpatient costs per episode	126,671.15	120,964.90	–	109,514.41	117,745.26	–	137,715.68	122,925.22	–
Outpatient									
Per visit (excluding pharmacy costs)									
Median (Q1, Q3)	568.50 (294.50, 1,028.00)	1,164.00 (335.00, 2,519.00)	<0.001^a^	545.00 (297.00, 995.00)	1,116.50 (335.00, 2,686.00)	0.004^a^	605.00 (292.00, 1,116.00)	1,265.00 (335.00, 2,347.00)	0.022^a^
Total outpatient visit costs	172,490	258,282	–	86,254	158,716	–	86,236	99,566	–
Pharmacy costs per visit									
Median (Q1, Q3)	497.00 (172.00, 1,325.00)	594.00 (192.50, 1,948.50)	0.221	464.00 (167.00, 1,166.00)	509.00 (155.00, 1,414.00)	0.322	637.00 (186.00, 1,524.50)	664.50 (244.00, 2,000.00)	0.311
Total outpatient pharmacy costs	155,721	183,938	–	61,598	76,311	–	94,123	107,627	–
Total outpatient costs^f^	328,211	442,220	–	147,852	235,027	–	180,359	207,193	–
Per episode									
Average outpatient visit costs	118.31	309.32	–	151.06	502.27	–	97.22	191.84	–
Average pharmacy costs	106.80	220.29	–	107.88	241.49	–	106.11	207.37	–
Average outpatient costs	225.11	529.61	–	258.94	743.76	–	203.34	399.22	–
ED									
Per visit									
Median (Q1, Q3)	6,745 (4,227, 11,249)	8,192 (4,487, 12,465)	0.198	6,157.50 (3,910.50, 9,987.50)	8,533.00 (5,324.00, 14,855.00)	0.052	6,985.00 (4,349.00, 11,688.00)	7,615.50 (3,994.50, 11,102.00)	0.404
Total ED costs	961,133	761,097	–	315,910	346,165	–	645,223	414,932	–
Average ED costs per episode	659.21	911.49	–	553.26	1,095.46	–	727.42	799.48	–
Total costs^g^	185,975,875	102,209,008	–	62,996,489	37,788,695	–	122,979,386	64,420,313	–
Average total costs per episode	127,555.47	122,406.00	–	110,326.60	119,584.48	–	138,646.43	124,123.92	–

a *p* value <0.05.

b Number of inpatient visits is defined as the number of IPD-associated hospital admissions.

c Total inpatient hospitalization is defined as the total duration of stay (in days) for all IPD-associated hospitalizations.

d Number of outpatient visits is defined as the number of hospital outpatient visits that occur on separate dates. Multiple records on the same date will be considered as a single visit.

e Number of ED visits are defined as the number of IPD-associated visits to the hospital ED that occur on separate dates.

f Total outpatient costs is defined by the sum of total outpatient visit costs and total outpatient pharmacy costs.

g Total costs is defined by the sum of total inpatient costs, total outpatient costs, and total ED costs.

h *p* value not computed for average costs per episode as they were calculated using the total costs divided by the number of episodes.

Abbreviations: ED, emergency department; IPD, invasive pneumococcal disease; PCV13, 13-valent pneumococcal conjugate vaccine; Q1, 25th percentile; Q3, 75th percentile; SD, standard deviation.

**Table 4.** Pneumonia-associated HCRU and costs during pre-PCV13 and post-PCV13 periods, by age groups Table 4. long description.

	≥ 18 years			18 to < 65 years			≥ 65 years		
	Pre-PCV13 (*n* = 3,632)	Post-PCV13 (*n* = 2,304)	*p* value	Pre-PCV13(*n* = 1,435)	Post-PCV13 (*n* = 866)	*p* value	Pre-PCV13(*n* = 2,197)	Post-PCV13(*n* = 1,438)	*p* value
**Healthcare resource utilization**									
Inpatient									
Number of visits^b^, *n*	4,021	2,631	–^h^	1,516	923	–	2,505	1,708	–
Length of hospitalization per visit (days)									
Median (Q1, Q3)	9 (5, 15)	8 (6, 15)	0.422	7 (5, 11)	7 (5, 12)	0.192	10 (6, 18)	10 (6, 17)	0.205
Total length of hospitalization^c^	49,913	31,714	–	15,520	9,077	–	34,393	22,637	–
Per episode									
Average number of visits	1.11	1.14	–	1.06	1.07	–	1.14	1.19	–
Average length of hospitalization (days)	13.74	13.77	–	10.82	10.48	–	15.66	15.74	–
Outpatient									
Number of visits^d^, *n*	2,424	1,529	–	1,117	645	–	1,307	884	–
Average number of visits per episode	0.67	0.66	–	0.78	0.75	–	0.60	0.62	–
ED									
Number of visits^e^, *n*	928	466	–	332	168	–	596	298	–
Average number of visits per episode	0.26	0.20	–	0.23	0.19	–	0.27	0.21	–
**Costs**									
Inpatient									
Per visit									
Median (Q1, Q3)	36,713.00 (20,517.00, 80,886.00)	37,157.00 (21,491.50, 77,926.00)	0.346	27,494.00 (16,492.00, 53,027.00)	27,948.50 (17,803.00, 56,080.00)	0.094	45,680.00 (25,039.00, 102,913.00)	43,099.50 (24,692.00, 96,950.00)	0.186
Total inpatient costs	304,686,949	187,589,620	–	89,173,818	50,970,861	–	215,513,131	136,618,759	–
Average inpatient costs per episode	83,889.58	81,419.11	–	62,142.03	58,857.81	–	98,094.28	95,006.09	–
Outpatient									
Per visit (excluding pharmacy costs)									
Median (Q1, Q3)	570 (367, 1,175)	656 (359, 1,318)	0.024^a^	540.00 (387.00, 1,106.00)	569.00 (380.00, 1,244.00)	0.158	594.00 (320.00, 1,223.00)	701.50 (336.50, 1,361.50)	0.034^a^
Total outpatient visit costs	1,469,921	1,065,331	–	715,116	445,473	–	754,805	619,858	–
Pharmacy costs per visit									
Median (Q1, Q3)	409.00 (127.50, 1,214.50)	332.50 (133.50, 865.50)	0.028^a^	258.50 (102.00, 791.00)	273.00 (106.00, 580.00)	0.305	638.50 (192.50, 1,710.50)	409.00 (171.00, 1,028.00)	0.001^a^
Total outpatient pharmacy costs	1,268,447	644,166	–	474,989	202,889	–	793,458	441,277	–
Total outpatient costs^f^	2,738,368	1,709,497	–	1,190,105	648,362	–	1,548,263	1,061,135	–
Per episode									
Average outpatient visit costs	404.71	462.38	–	498.34	514.40	–	343.56	431.06	–
Average pharmacy costs	349.24	279.59	–	331.00	234.28	–	361.16	306.87	–
Average outpatient costs	753.96	741.97	–	829.34	748.69	–	704.72	737.92	–
ED									
Per visit									
Median (Q1, Q3)	4,704.50 (3,307.50, 7,660.50)	5,142.00 (3,635.00, 8,493.00)	0.017^a^	4,057.00 (2,995.00, 6,346.50)	4,411.00 (3,507.00, 6,533.50)	0.079	5,086.00 (3,651.00, 8,286.50)	5,765.00 (3,814.00, 8,900.00)	0.040^a^
Total ED costs	5,147,554	2,900,277	–	1,740,233	923,495	–	3,407,321	1,976,782	–
Average ED costs per episode	1,417.28	1,258.80	–	1,212.71	1,066.39	–	1,550.90	1,374.68	–
Total costs^g^	312,572,871	192,199,394	–	92,104,156	52,542,718	–	220,468,715	139,656,676	–
Average total costs per episode	86,060.81	83,419.88	–	64,184.08	60,672.89	–	100,349.89	97,118.69	–

a *p* value <0.05.

b Number of inpatient visits is defined as the number of pneumonia-associated hospital admissions.

c Total inpatient hospitalization is defined as the total duration of stay (in days) for all pneumonia-associated hospitalizations.

d Number of outpatient visits is defined as the number of hospital outpatient visits that occur on separate dates. Multiple records on the same date will be considered as a single visit.

e Number of ED visits are defined as the number of pneumonia-associated visits to the hospital ED that occur on separate dates.

f Total outpatient costs is defined by the sum of total outpatient visit costs and total outpatient pharmacy costs.

g Total costs is defined by the sum of total inpatient costs, total outpatient costs, and total ED costs.

h *p* value not computed for average costs per episode as they were calculated using the total costs divided by the number of episodes.

Abbreviations: ED, emergency department; PCV13, 13-valent pneumococcal conjugate vaccine; Q1, 25th percentile; Q3, 75th percentile; SD, standard deviation.

### Sensitivity analyses

When disease episodes were identified using both pneumococcal-specific and non-specific codes, baseline IPD IR was found to be significantly lower in the post-PCV13 period (IRR = 0.84; 95% CI: [0.83, 0.85]). This was accompanied by a significant decrease in the IPD IR trend (IRR = 0.98; 95% CI: [0.98, 0.98]), which did not mirror the findings of the primary analyses. For pneumonia, a significantly higher baseline IR was observed in the post-PCV13 period (IRR = 1.31; 95% CI: [1.30, 1.32]), which was also different from that observed in the primary analyses. There was also a statistically significant marginal increase in the pneumonia IR trend (IRR = 1.00; 95% CI: [1.00, 1.00]). The figures for sensitivity analyses are shown in Supplementary Figures 1 and 2.

## Discussion

To our knowledge, this is the first study in Taiwan to quantify both the clinical and economic burden associated with IPD and pneumonia in adults using the NHIRD, before and following the inclusion of PCV13 in the CIP.

The ITSA results showed that baseline IR was significantly lower in the post-PCV13 period for both IPD and pneumonia compared with the pre-PCV13 period, especially in the elderly ≥65 years. This observation may be attributed to the increased uptake of PPSV23 among older adults [19, 20]. With financial support from a non-governmental organization, PPSV23 has been provided to Taiwanese individuals aged ≥75 years through a pilot programme since 2007, and has been extended to all elderly since 2008 [8]. Hence, the use of PPSV23 has increased steadily in Taiwan, with the cumulative coverage rate among those aged ≥75 years almost doubling from 12% in 2007 to 20.8% in 2018, leading to a decreasing trend of IRs observed in the pre-PCV13 period and a significantly lower baseline IR observed in the post-PCV13 period [21, 22].

In addition to the increased uptake of PPSV23, the significant reduction in IR of IPD and pneumonia among adults in the post-PCV13 period of our study suggests a herd protective effect [9]. The herd effect from childhood vaccination has been reported in Australia, the United Kingdom, and the United States after the incorporation of PCV7 into their national CIPs [23–25]. In Taiwan, earlier studies have also shown a reduction in IPD incidence among adults after PCV13 introduction [26–28]. In a study utilizing national data from the Taiwan Centers for Disease Control (CDC), it was estimated that the PCV13 catch-up vaccination programme decreased IPD IR by 69% in children, but more modestly by 39% in elderly ≥70 years from 2012 to 2017 [26].

Serotype replacement could possibly explain the increase in IR trends for both IPD and pneumonia in the post-PCV13 period of our study. Replacement of vaccine serotypes by non-vaccine serotypes (NVTs) is caused by selection pressure following universal PCV immunization, and this could nullify the benefit from decreased prevalence of vaccine serotypes [29]. Research conducted before and after the introduction of PCV13 has found that the leading serotypes in Taiwan have been gradually replaced with other NVTs [30, 31]. Based on a surveillance report from the Taiwan CDC, the proportion of NVTs, (e.g., 15A, 23A, and 35B) had been increasing among IPD isolates from adults ≥65 years during 2008–2016 [30]. Hence, the increasing prevalence of NVTs could have led to the rebound of pneumococcal IRs in our study.

Sensitivity analyses were largely consistent with our primary analyses. However, contrary to the primary analyses, our ITSA showed that baseline pneumonia IR was significantly higher among adults in the post-PCV13 period, compared with the pre-PCV13 period. It is most likely that the sensitivity analyses overestimated the incidence of pneumonia. When ICD codes for non-specific and all-cause pneumonia were used, the number of pneumonia episodes identified increased considerably from 7,574 to 946,764 over the study period (2011–2019). Hence, the proportion of pneumococcal-specific episodes became a very small proportion of all episodes (<1%) in the sensitivity analyses. This means that any change in the number of non-specific episodes will affect the overall pneumonia IR and trend to a greater extent. In addition, the higher number of non-specific episodes in the post-PCV13 period and the rise of pneumonia hospitalizations may be attributed to the outbreak of influenza in Taiwan (2016–2017) [32].

The results from our study showed that there were more deaths associated with IPD, compared with pneumonia. Our estimate for IPD CFR was 17.23% in the overall population, similar to a previous study which reported an IPD CFR of 18.2% between 2008 and 2013 [33]. The findings from our study also revealed that CFR for both IPD and pneumonia increased with age. This was generally in line with other studies, which reported higher IPD mortality in the older age groups [33–35].

The estimated HCRU and costs per disease episode in our study were generally higher in IPD than in pneumonia, but total costs were the highest for pneumonia, indicating a larger impact on healthcare burden. Consistent with a previous study, there were also considerable HCRU and costs associated with both IPD and pneumonia in the elderly ≥65 years [35]. This finding could be due to several reasons. Firstly, adults aged ≥65 years commonly have multiple risk factors for pneumococcal infection, which can increase the risk of more severe disease and poorer outcomes [36]. Secondly, the exacerbation of underlying conditions and the development of complications could also lead to additional HCRU and costs [36]. Thirdly, as the majority of overall costs consisted of inpatient costs, total costs would be strongly correlated with disease severity and length of hospitalization, leading to greater economic burden in the elderly.

There are several limitations to the study. It is possible that there was an underestimation of disease incidence and burden, as the disease episodes were identified using pneumococcal-specific ICD codes. The accuracy of these codes has not been validated due to the lack of laboratory or microbial culture results in the NHIRD. On the other hand, sensitivity analyses conducted using both pneumococcal-specific and non-specific ICD codes may result in an overestimation. In addition, as NHIRD is a national claims database, self-pay visits or out-of-pocket expenses were not captured. However, since treatment for IPD and pneumonia is reimbursed under the National Health Insurance system in Taiwan, it is expected that the number of self-paying patients is small.

## Conclusion

This study provided a comprehensive assessment of the burden of hospitalized pneumococcal disease among adults and its association with the inclusion of PCV13 in Taiwan’s CIP. IRs of hospitalized IPD and pneumonia decreased significantly after the introduction of PCV13 into the CIP. However, clinical and economic burden remained high, especially in the elderly ≥65 years. Further research is needed to investigate the cause of the increasing trend in IRs of hospitalized IPD and pneumonia in the post-PCV13 era. Continuous surveillance in Taiwan is also important to update local epidemiological information, detect emerging serotypes, and generate knowledge for future vaccine development and policy.

## Supporting information

10.1017/S0950268826101411.sm001 Wang et al. supplementary material 1 Wang et al. supplementary material

10.1017/S0950268826101411.sm002 Wang et al. supplementary material 2 Wang et al. supplementary material

## Data Availability

The datasets generated and/or analysed during the current study are not publicly available. The data analysed in this study were obtained from the Taiwan’s National Health Insurance Research Database, which is provided by the National Health Insurance Administration and maintained by the Health and Welfare Data Science Center (https://dep.mohw.gov.tw/DOS/cp-5119-59201-113.html), Ministry of Health and Welfare, Executive Yuan, Taiwan. The release of the claims dataset is prohibited by the Taiwanese government. Additional details on International Classification of Diseases codes used in this study are available in Additional File 1.

## Supplementary material

The supplementary material for this article can be found at http://doi.org/10.1017/S0950268826101411.

## Long descriptions

### Table 1 Long description

The table is structured with seven columns. The first column lists the Condition and age groups. The next three columns fall under the header Baseline I R, and the final three columns fall under the header Trend of I R. Both sections include sub-headers for I R R, 95 percent C I, and p value.

Under the I P D condition:

* For age 18 years and older, Baseline I R R is 0.76 (C I 0.63 to 0.93, p 0.007) and Trend I R R is 1.02 (C I 1.00 to 1.03, p 0.045).

* For age 18 to less than 65 years, Baseline I R R is 0.90 (C I 0.66 to 1.23, p 0.507) and Trend I R R is 1.04 (C I 1.01 to 1.06, p 0.010).

* For age 65 years and older, Baseline I R R is 0.68 (C I 0.53 to 0.87, p 0.002) and Trend I R R is 1.00 (C I 0.98 to 1.02, p 0.831).

Under the Pneumonia condition:

* For age 18 years and older, Baseline I R R is 0.87 (C I 0.77 to 0.98, p 0.019) and Trend I R R is 1.02 (C I 1.01 to 1.03, p less than 0.001).

* For age 18 to less than 65 years, Baseline I R R is 0.90 (C I 0.74 to 1.09, p 0.291) and Trend I R R is 1.03 (C I 1.01 to 1.04, p 0.002).

* For age 65 years and older, Baseline I R R is 0.83 (C I 0.71 to 0.97, p 0.016) and Trend I R R is 1.01 (C I 1.00 to 1.03, p 0.045).

Navigate back to Table 1.

### Figure 1 Long description

The vertical y axis is labeled Incidence rate per 100,000 person-years with a scale from 0 to 6 in increments of 0.5. The horizontal x axis is labeled Year with annual increments from 2011 to 2019. A legend in the top right identifies three groups.

* The group greater than or equal to 65 years is represented by a dotted line. It starts at approximately 5.5 in 2011, peaks at 5.7 in 2013, and shows a sharp decline after 2015, dropping to approximately 3.6 by 2016 and remaining between 3.3 and 3.7 through 2019.

* The group greater than or equal to 18 years is represented by a solid line. It fluctuates between 1.5 and 1.9 from 2011 to 2015, then drops to approximately 1.2 in 2016, ending at roughly 1.4 in 2019.

* The group 18 to less than 65 years is represented by a dashed line. It remains the lowest throughout the period, starting near 0.9 in 2011, peaking at 1.0 in 2015, and then dropping to approximately 0.6 for the remainder of the period from 2016 to 2019.

Navigate back to Figure 1.

### Figure 2 Long description

A multi-panel figure with three line graphs labeled a, b, and c. All graphs share an x-axis representing Year from 2011 to 2019 and a y-axis representing Incidence rate per 100,000 person-months from 0.0 to 1.0. Each graph is divided into three chronological sections: Pre-P C V 13 (2011 to late 2014), Transition (late 2014 to late 2015), and Post-P C V 13 (late 2015 to 2019). Solid lines show observed trends, and dashed lines show predicted trends with shaded confidence intervals.

* Panel a (Adults aged 18 years or older): The incidence rate fluctuates between 0.1 and 0.2. The predicted trend line shows a slight downward slope in the pre-period and a very slight upward slope in the post-period, starting from a lower baseline.

* Panel b (Adults aged 18 to less than 65 years): The incidence rate is lower, fluctuating between 0.0 and 0.15. The predicted trend line is nearly flat in the pre-period and shows a slight upward trajectory in the post-period.

* Panel c (Adults aged 65 years or older): This group shows the highest incidence, fluctuating between 0.2 and 0.6. The pre-period shows a downward predicted trend. The post-period begins at a significantly lower baseline around 0.3 and remains relatively stable with a slight downward slope.

Navigate back to Figure 2.

### Figure 3 Long description

The x-axis is labeled Year and ranges from 2011 to 2019. The y-axis is labeled Incidence rate per 100,000 person-years and ranges from 0 to 16 in increments of 2. A legend in the top right identifies three age groups.

* The group greater than or equal to 65 years is represented by a dotted line. It starts at approximately 14 in 2011, shows a fluctuating downward trend to a low of approximately 9 in 2017, peaks again at 11 in 2018, and ends at approximately 9 in 2019.

* The group greater than or equal to 18 years is represented by a solid line. It remains relatively stable, starting at approximately 4.5 in 2011 and gradually declining to approximately 3.5 by 2019.

* The group 18 to less than 65 years is represented by a dashed line. It is the lowest data series, starting at approximately 2.2 in 2011 and ending at approximately 1.8 in 2019, with a slight dip in 2017.

Navigate back to Figure 3.

### Figure 4 Long description

The figure consists of three panels, each with an X-axis representing the Year from 2011 to 2019 and a Y-axis representing the Incidence rate per 100,000 person-months ranging from 0.0 to 2.0. Each graph is divided into three horizontal sections: Pre-P C V 13 (2011 to mid-2014), Transition (mid-2014 to late 2015), and Post-P C V 13 (late 2015 to 2019). Solid lines show observed trends with seasonal fluctuations, and dashed lines show predicted trends with shaded confidence intervals.

* Panel a (Adults aged 18 years or older): The incidence rate starts near 0.5 in 2011 and shows a slight downward trend during the Pre-P C V 13 period. In the Post-P C V 13 period, the baseline is lower, starting near 0.3, with a very gradual upward slope in the predicted trend line.

* Panel b (Adults aged 18 to less than 65 years): The incidence rate is the lowest of the three groups, starting below 0.5. The Pre-P C V 13 and Post-P C V 13 periods show similar baseline levels, with the Post-P C V 13 period exhibiting a slight increase in the predicted trend over time.

* Panel c (Adults aged 65 years or older): This group shows the highest incidence rates. In the Pre-P C V 13 period, the rate starts near 1.4 and trends downward toward 1.0. Following the transition, the Post-P C V 13 period begins at a significantly lower baseline of approximately 0.8, with a stable or slightly increasing predicted trend through 2019.

Navigate back to Figure 4.

### Table 2 Long description

The table is divided into two main sections: I P D (Invasive Pneumococcal Disease) and Pneumonia. Each section provides data for three age groups: 18 years and older, 18 to less than 65 years, and 65 years and older. Data is categorized into four main columns: Overall, Pre-P C V 13 period, Transition, and Post-P C V 13 period. Each category includes Cases (N), Deaths (n), and C F R (percent).

For I P D:

* 18 years and older: Overall C F R is 17.23 percent (3,001 cases, 517 deaths). Post-P C V 13 C F R decreased to 16.17 percent.

* 18 to less than 65 years: Overall C F R is 9.31 percent. Post-P C V 13 C F R decreased to 7.89 percent.

* 65 years and older: Overall C F R is 22.30 percent. Post-P C V 13 C F R decreased to 21.22 percent.

For Pneumonia:

* 18 years and older: Overall C F R is 7.00 percent (7,574 cases, 530 deaths). Post-P C V 13 C F R decreased to 5.56 percent.

* 18 to less than 65 years: Overall C F R is 3.34 percent. Post-P C V 13 C F R decreased to 2.55 percent.

* 65 years and older: Overall C F R is 9.31 percent. Post-P C V 13 C F R decreased to 7.40 percent.

Abbreviations used: C F R (case fatality rates), I P D (invasive pneumococcal disease), P C V 13 (13-valent pneumococcal conjugate vaccine).

Navigate back to Table 2.

### Table 3 Long description

The table is divided into two main sections: Healthcare resource utilization and Costs. Data is segmented into three age groups: 18 years or older, 18 to less than 65 years, and 65 years or older. Each group is further split into Pre-P C V 13 and Post-P C V 13 periods with associated p-values.

Healthcare resource utilization section:

* Inpatient visits: For the 18 years or older group, visits decreased from 1,561 to 894. Median length of stay changed from 12 to 11 days. For the 65 years or older group, the median length of stay decreased from 13 to 11 days with a significant p-value of 0.016.

* Outpatient visits: Average visits per episode increased across all groups, for example, from 0.19 to 0.28 in the 18 years or older group.

* E D visits: Average visits per episode showed slight increases across all age cohorts.

Costs section:

* Inpatient costs: Median cost per visit for the 18 to less than 65 group increased significantly from 54,216.00 to 65,364.00 (p-value 0.006). Total inpatient costs for the 18 years or older group dropped from 184,686,531 to 101,005,691.

* Outpatient costs: Median costs per visit increased significantly across all groups (p-values less than 0.05), nearly doubling in most cases. For the 18 years or older group, it rose from 568.50 to 1,164.00.

* Total costs: For the 18 years or older group, total costs decreased from 185,975,875 to 102,209,008. Average total costs per episode remained relatively stable, changing from 127,555.47 to 122,406.00.

Navigate back to Table 3.

### Table 4 Long description

The table is divided into three main age cohorts: 18 years or older, 18 to less than 65 years, and 65 years or older. Each cohort is split into Pre-P C V 13 and Post-P C V 13 periods with associated p values.

Healthcare Resource Utilization section:

- Inpatient visits: For the 18 or older group, visits decreased from 4,021 to 2,631. Median length of stay remained stable at 9 and 8 days respectively.

- Outpatient visits: For the 18 or older group, visits decreased from 2,424 to 1,529.

- E D visits: For the 18 or older group, visits decreased from 928 to 466.

Costs section (values in currency units):

- Inpatient costs per visit: Median for the 18 or older group was 36,713.00 pre-P C V 13 and 37,157.00 post-P C V 13.

- Total inpatient costs: Decreased from 304,686,949 to 187,589,620 for the 18 or older group.

- Outpatient costs per visit: Median increased significantly for the 65 or older group from 594.00 to 701.50 (p = 0.034).

- Pharmacy costs per visit: Median decreased significantly for the 65 or older group from 638.50 to 409.00 (p = 0.001).

- E D costs per visit: Median increased for the 18 or older group from 4,704.50 to 5,142.00 (p = 0.017).

- Total costs: For the 18 or older group, total costs fell from 312,572,871 to 192,199,394. Average total costs per episode decreased from 86,060.81 to 83,419.88.

Navigate back to Table 4.
