# Supplementary material for: Association between introduction of 13-valent pneumococcal conjugate vaccine and burden of hospitalized adult pneumococcal disease in Taiwan – a retrospective database study
Source: Epidemiol Infect. 2026 Jun 26;154:e103. doi: 10.1017/S0950268826101411 (PMC13437988; doi:10.1017/S0950268826101411)
Supplement: Supplementary file 1 [file S0950268826101411sup001.docx]

**Supplementary Table 1**

**International Classification of Diseases, Ninth and Tenth revisions (ICD-9 and ICD-10) codes for invasive pneumococcal disease and pneumonia**

| **Disease** | **Category** | **ICD9** | **ICD10** | **Description** |
| --- | --- | --- | --- | --- |
| IPD | Pneumococcal-specific | 038.2;  038.0+041.2;  038.9+041.2;  790.7+041.2;  320.1;  320.2+041.2;  320.8x/320.9 +041.2;  322.9+041.2  510.x+041.2;  511.1/511.9 +041.2;  038.2+ACP code;  038.0+041.2+ACP code;  038.9+041.2+ACP code;  790.7+041.2+ACP code;  038.0+481;  038.9+481;  790.7+481;  041.2;  567.1;  420.9x+041.2;  421.0+041.2;  421.1/421.9 +041.2;  567.23+041.2;  730.0x, 730.2x +041.2;  711.0x/711.9x +041.2 | A40.3;  A40.9+B95.3;  A41.9+B95.3;  R78.81+B95.3;  G00.1;  G00.2+B95.3;  G00.8/G00.9 +B95.3;  G03.9+B95.3;  J86.x+B95.3;  J90/J91.8+B95.3;  A40.3+ACP code;  A40.9+B95.3+ACP code;  A41.9+B95.3+ACP code;  R78.81+B95.3+ACP code;  A40.9+J13;  A41.9+J13;  R78.81+J13;  M00.1x;  K65.8+B95.3;  I30.1+B95.3;  I33.0+B95.3;  I33.9 +B95.3;  K65.2+B95.3;  M86.1x/M86.2x/M86.9 +B95.3;  M00.0x, M00.2x, M00.8x, M00.9+B95.3 | Pneumococcal septicemia  Streptococcal septicemia + PNI  Unspecified septicemia + PNI  Bacteremia + PNI  Pneumococcal meningitis  Streptococcal meningitis + PNI  Bacterial meningitis, unspecified + PNI  Meningitis, unspecified + PNI  Empyema + PNI  Pleural effusion + PNI  Pneumococcal arthritis  Pneumococcal peritonitis  Infective pericarditis + PNI  Bacterial endocarditis + PNI  Endocarditis, unspecified + PNI  Spontaneous bacterial peritonitis + PNI  Osteomyelitis + PNI  Pyogenic/unspecified arthritis + PNI |
|  | Non-specific | 038.0;  038.9;  790.7;  320.2;  320.8x/320.9;  322.9;  510.x;  511.1/511.9;  038.0+ACP code^a^;  038.9+ACP code^a^;  790.7+ACP code^a^  420.9;  421.0;  421.1/421.9;  567.23;  730.0x, 730.2x;  711.0x/711.9x | A40.9;  A41.9;  R78.81;  G00.2;  G00.8/G00.9;  G03.9;  J86.x;  J90/J91.8;  A40.9+ACP code ^a^;  A41.9+ACP code ^a^;  R78.81+ACP code ^a^;  I30.1;  I33.0;  I33.9;  K65.2;  M86.1x/M86.2x/M86.9;  M00.0x, M00.2x, M00.8x, M00.9 | Streptococcal septicemia  Unspecified septicemia  Bacteremia  Streptococcal meningitis  Bacterial meningitis, unspecified  Meningitis, unspecified  Empyema  Pleural effusion  Infective pericarditis  Bacterial endocarditis  Endocarditis, unspecified  Spontaneous bacterial peritonitis  Acute or unspecified osteomyelitis  Pyogenic/unspecified arthritis |
| Pneumonia | Pneumococcal-specific | 481;    482.9+041.2;  485+041.2;  486+041.2 | J13;  J18.1+B95.3;  J15.x+B95.3;  J18.0+B95.3;  J18.9+B95.3 | Pneumococcal pneumonia  Lobar pneumonia, unspecified + PNI  Bacterial pneumonia, unspecified+ PNI  Bronchopneumonia + PNI  Pneumonia, organism unspecified + PNI |
|  | Non-specific | 482.9;  485;  486 | J15.x/J18.1;  J18.0;  J18.9 | Bacterial pneumonia, unspecified  Bronchopneumonia  Pneumonia, organism unspecified |
|  | All-cause pneumonia (ACP) | 480.x (480.0-480.3, 480.8, 480.9);    481;  482.x;  483.x (483.0, 483.1, 483.8);  484.x (484.1, 484.3, 484.5-484.8);  485;  486;  487.0 | J12.x (J12.0, J12.1, J12.2, J12.3, J12.81, J12.89, J12.9);  J13, J18.1;  A48.1, J14, J15.0, J15.1, J15.2x (J15.20, J15.211, J15.212, J15.29), J15.3, J15.4, J15.5, J15.6, J15.8, J15.9;  J15.7, J16.x (J16.0, J16.8);  A22.1, A37.91, B25.0, B44.0, J17;    J18.0;  J18.2, J18.8, J18.9;  J11.0x (J11.00, J11.08) | Viral pneumonia  Pneumococcal pneumonia  Other bacterial pneumonia  Pneumonia due to other specified organism  Pneumonia in infectious diseases classified elsewhere  Bronchopneumonia, organism unspecified  Pneumonia, organism unspecified  Influenza with pneumonia |

Abbreviations: ACP, All-cause pneumonia; IPD, invasive pneumococcal disease; PNI, Pneumococcal infection.

**Supplementary Table 2**

**Incidence rates of hospitalized invasive pneumococcal disease and pneumonia in adults from 2011-2019, by age groups**

| **Year** | **Total (≥ 18 years)** | | |  | **18 to < 65 years** | | |  | **≥ 65 years** | | |
| --- | --- | --- | --- | --- | --- | --- | --- | --- | --- | --- | --- |
|  | **Episodes** | **Person-years** | **Incidence rate per 100,000 person-years** |  | **Episodes** | **Person-years** | **Incidence rate per 100,000 person-years** |  | **Episodes** | **Person-years** | **Incidence rate per 100,000 person-years** |
| **IPD** | | |  |  |  |  |  |  |  |  |  |
| 2011-2019 | 3,001 | 194,781,797.33 | 1.54 |  | 1,171 | 153,618,305.06 | 0.76 |  | 1,830 | 41,163,492.27 | 4.45 |
| 2011 | 381 | 20,979,833.58 | 1.82 |  | 158 | 16,947,772.47 | 0.93 |  | 223 | 4,032,061.12 | 5.53 |
| 2012 | 350 | 21,219,564.17 | 1.65 |  | 147 | 17,091,560.90 | 0.86 |  | 203 | 4,128,003.27 | 4.92 |
| 2013 | 384 | 21,341,278.88 | 1.80 |  | 144 | 17,114,517.19 | 0.84 |  | 240 | 4,226,761.69 | 5.68 |
| 2014 | 343 | 21,513,189.64 | 1.59 |  | 122 | 17,151,533.96 | 0.71 |  | 221 | 4,361,655.68 | 5.07 |
| 2015 | 405 | 21,682,660.38 | 1.87 |  | 169 | 17,175,182.63 | 0.98 |  | 236 | 4,507,477.75 | 5.24 |
| 2016 | 268 | 21,878,752.02 | 1.23 |  | 99 | 17,180,970.33 | 0.58 |  | 169 | 4,697,781.69 | 3.60 |
| 2017 | 270 | 21,933,023.63 | 1.23 |  | 108 | 17,052,478.52 | 0.63 |  | 162 | 4,880,545.12 | 3.32 |
| 2018 | 297 | 22,056,119.51 | 1.35 |  | 109 | 16,989,813.04 | 0.64 |  | 188 | 5,066,306.47 | 3.71 |
| 2019 | 303 | 22,177,375.53 | 1.37 |  | 115 | 16,914,476.03 | 0.68 |  | 188 | 5,262,899.50 | 3.57 |
| **Pneumonia** | |  |  |  |  |  |  |  |  |  |  |
| 2011-2019 | 7,574 | 194,781,797.33 | 3.89 |  | 2,935 | 153,618,305.06 | 1.91 |  | 4,639 | 41,163,492.27 | 11.27 |
| 2011 | 938 | 20,979,833.58 | 4.47 |  | 375 | 16,947,772.47 | 2.21 |  | 563 | 4,032,061.12 | 13.96 |
| 2012 | 923 | 21,219,564.17 | 4.35 |  | 377 | 17,091,560.90 | 2.21 |  | 546 | 4,128,003.27 | 13.23 |
| 2013 | 930 | 21,341,278.88 | 4.36 |  | 352 | 17,114,517.19 | 2.06 |  | 578 | 4,226,761.69 | 13.68 |
| 2014 | 841 | 21,513,189.64 | 3.91 |  | 331 | 17,151,533.96 | 1.93 |  | 510 | 4,361,655.68 | 11.69 |
| 2015 | 847 | 21,682,660.38 | 3.91 |  | 325 | 17,175,182.63 | 1.89 |  | 522 | 4,507,477.75 | 11.58 |
| 2016 | 731 | 21,878,752.02 | 3.34 |  | 298 | 17,180,970.33 | 1.73 |  | 433 | 4,697,781.69 | 9.22 |
| 2017 | 702 | 21,933,023.63 | 3.20 |  | 254 | 17,052,478.52 | 1.49 |  | 448 | 4,880,545.12 | 9.18 |
| 2018 | 871 | 22,056,119.51 | 3.95 |  | 314 | 16,989,813.04 | 1.85 |  | 557 | 5,066,306.47 | 10.99 |
| 2019 | 791 | 22,177,375.53 | 3.57 |  | 309 | 16,914,476.03 | 1.83 |  | 482 | 5,262,899.50 | 9.16 |
| Abbreviations: IPD, invasive pneumococcal disease. | | | | | | | | | | | |
