# Supplementary material for: Association between introduction of 13-valent pneumococcal conjugate vaccine and burden of hospitalized adult pneumococcal disease in Taiwan – a retrospective database study
Source: Epidemiol Infect. 2026 Jun 26;154:e103. doi: 10.1017/S0950268826101411 (PMC13437988; doi:10.1017/S0950268826101411)
Supplement: Supplementary file 2 [file S0950268826101411sup002.docx]

**Supplementary Figures**

**Supplementary Figure 1: Trend of quarterly incidence rates of hospitalized invasive pneumococcal disease in adults from 2011-2019 – sensitivity analyses**

**Pre-PCV13**

**Transition**

**Post-PCV13**

**Supplementary Figure 2: Trend of quarterly incidence rates of hospitalized pneumonia in adults from 2011-2019 – sensitivity analyses**

**Post-PCV13**

**Transition**

**Pre-PCV13**
